# Traditional vs. Automated Computer Image Analysis—A Comparative Assessment of Use for Analysis of Digital SEM Images of High-Temperature Ceramic Material

**DOI:** 10.3390/ma16020812

**Published:** 2023-01-13

**Authors:** Ilona Jastrzębska, Adam Piwowarczyk

**Affiliations:** 1 Faculty of Materials Science and Ceramics, AGH University of Science and Technology in Cracow, al. A. Mickiewicza 30, 30-059 Cracow, Poland; 2 Faculty of Mechanical Engineering, Cracow University of Technology, al. Jana Pawła II 37, 31-864 Cracow, Poland

**Keywords:** ceramic, spinel, copper, SEM/EDS, digital image, computer analysis, stereology

## Abstract

Image analysis is a powerful tool that can be applied in scientific research, industry, and everyday life, but still, there is more room to use it in materials science. The interdisciplinary cooperation between materials scientists and computer scientists can unlock the potential of digital image analysis. Traditional image analysis used in materials science, manual or computer-aided, permits for the quantitative assessment of the coexisting components at the cross-sections, based on stereological law. However, currently used cutting-edge tools for computer image analysis can greatly speed up the process of microstructure analysis, e.g., via simultaneous extraction of quantitative data of all phases in an SEM image. The dedicated digital image processing software *Aphelion* was applied to develop an algorithm for the automated image analysis of multi-phase high-temperature ceramic material. The algorithm recognizes each phase and simultaneously calculates its quantity. In this work, we compare the traditional stereology-based methods of image analysis (linear and planimetry) to the automated method using a developed algorithm. The analysis was performed on a digital SEM microstructural image of high-temperature ceramic material from the Cu-Al-Fe-O system, containing four different phase components. The results show the good agreement of data obtained by classical stereology-based methods and the developed automated method. This presents an opportunity for the fast extraction of both qualitative and quantitative from the SEM images.

## 1. Introduction

Image analysis is becoming an increasingly used tool for various scientific research [1,2,3,4,5,6], industrial [7,8] and medical [9,10] applications. Often, we are unaware that image analysis accompanies us in our daily lives. Examples of this are traffic analysis, control of vehicle speed, and the detection of license plates [11], or pedestrian detection [12], which all point to smart city development [13]. Other examples of important industrial applications are monitoring the quality of rotors in wind turbines, which allows for early fault detection [7,8], and biometric security [14,15]. The development of image recognition algorithms by automated methods such as in [16] can greatly increase the accuracy of images and obtain plausible results. In particular, the application of image analysis algorithms allows for significantly enhanced diagnostic methods in medicine. An example is the application of a phase retrieval algorithm in an imaging method coupled with computer tomography (CT), which improved the visibility of weakly absorbing objects, thus permitting a lower radiation dose without loss of image quality [10]. Another example is using the 2D phase contrast algorithm, which retrieved better X-ray images with additional information. Moreover, coupling image detection algorithms with methods using high-energy sources, such as synchrotron radiation (e.g., SOLARIS [6]), or facilities with a thermal neutron beam (e.g., VIVALDI [17]) have recently allowed profound progress in the diagnostics of tumors in humans [18] and large-scale research facilities have been utilized as neutron research reactor facilities with image-plate detectors for crystallography and biology applications [19,20]. This has an extremely positive impact on both medical and industrial imaging. In this way, image analysis algorithms have been developed to support progress into safer and more sustainable development of nations. 

Image analysis is also a powerful tool in materials science. Many instances of its successful use have been reported so far. Digital image processing in joining technology permitted detection of the border between the melting pool of liquid metal and adjacent non-molten base metal despite their temperature being the same, as they are at the phase transformation stage [1]. This research afforded the opportunity to develop a smart welding filter equipped with augmented reality, which displays additional information to the operator, making the joining process more precise, operator-friendly, and effective.

In materials engineering, microstructure mostly determines materials’ properties. Modifications on the microstructural scale enable tailoring of the desired properties of a material. Scanning electron microscopy equipped with energy (SEM/EDS) or electron microprobe analysis with weave dispersive spectroscopy (EPMA/WDS) are common and effective tools that permit the extraction of spectroscopic qualitative information [21]. The obtained microstructural images can be subjected to image analysis to retrieve more data on materials, such as the quantity of the individual phase components. Traditional methods based on stereology rules can be applied, but they are time-consuming and less accurate due to the occurrence of systematic errors. The application of automated, computer-based methods has been recently developed for different kinds of materials. Binarization was used to detect the pores in cement pastes with different W/C ratios and their further geometrical characterization (diameter, roundness) to investigate the formation of capillary pores [4]. Image recognition and analysis were conducted on Al_2_O_3_/WS_2_ coatings on Al alloys to determine the volume share of areas of distinguishing filamentous morphology [5]. Kubinova et al. [22] compared several stereological and digital methods for estimating the surface area and volume of cells on confocal microscopy images and discussed their pros and cons in the context of the isotropy of the studied material. 

Image analysis can be performed on different types of photographs. Different attributes of every individual phase on an image can be used for the development of a robust algorithm for color, shape, or area. The image analysis covers object detection (qualitative information) as well as the measurement of the amounts of specific objects (quantitative information). The human is the “link” in the analysis process and has control over the program action, so also impacts the accuracy of the results. If input data (an image or a series of images) are prepared inadequately in the initial stage, the output data will be erroneous, inaccurate, and overestimated or underestimated. 

**Figure 1:**
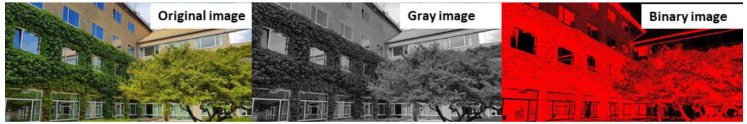
Comparison of an original color image with grey and binary images. Aarhus University campus, during 15th International Congress for Stereology and Image Analysis, Denmark 2019.

Comparison of traditional (manual/computer-aided) to automated computer measurements pros and cons can be performed. A computer will not count anything on its own, while a human must control a machine. Based on knowledge and experience, a human must precisely determine what shall be counted and assess whether the result is correct. As measurements are performed after binarization of the original image (Figure 1), preceded by various operations, a representative and good-quality image is of crucial significance.

One of the most common problems in image analysis is uneven background, which derives from the unequal lightening of the objects. This occurs especially in optical microscopy and may be a serious problem in appropriate object recognition, especially for low-contrast images. Often, it is not visible to the human eye, which leads to unreliable quantification and misinterpretation. A new method for shade correction in optical microscopy images, developed by Gądek et al. [23], was based on the simulation of the image background in which pixel values represent smooth grey-level changes. In contrast, Biżantowicz [24] developed a focus stacking algorithm for SEM images, which eliminates the adverse features of SEM photos, such as drifts or changes in geometry, and allowed for an increase in the depth of field (DoF) via digital image correction combined with assembling a series of photographs into one image.

Currently, in the characterization of ceramic materials, the most common technique of microstructural analysis is the SEM/EDS technique, which is widely available both at universities and in numerous R&D departments of industrial companies. The method requires a small sample of about 1 cm^2^ area and provides a wide range of enlargements. However, mostly qualitative information is retrieved from these images, while quantitative data are determined from manual or computer-aided calculations based on stereology laws, or, using complementary methods, e.g., refinement of X-ray patterns; these are both, in fact, laborious and time-consuming. In this work, we compare classical stereology-based vs. automated computer analysis of grey SEM microstructural images of high-temperature ceramic material from the Cu-Al-Fe-O system. Starting from showing versatile transformations, which enable increased image quality, we finally present the algorithm permitting for retrieving reliable quantitative data on the amounts of the microstructural phase objects present in the analyzed material. The application of this kind of algorithm can greatly enhance the extraction of additional data from a single SEM image; thus, it can make R&D activities more effective and sustainable.

## 2. Methods and Materials

The material taken for the image analysis was ceramic oxide material, produced via the arc melting technique [25] that was previously used successfully for the synthesis of numerous high-temperature spinel compounds [26,27,28] and high-temperature materials [29,30]. The starting materials were analytical grade powders of Fe_2_O_3_, CuO, and Al_2_O_3_ (Sigma Aldrich) mixed in the molar proportions 0.25:0.5:1 and homogenized in a ball mill. The sample for arc-melting was prepared in the form of a cylindrical-shaped disc of 20 mm diameter and 10 mm height. The arc-melted material was observed using a scanning electron microscope (Nova NanoSEM200 (FEI)) equipped with an energy-dispersive spectrometer (EDX). The imaging was performed in back-scattered electron (BSE) mode, using an accelerating voltage of 18 kV and 2000× magnification.

Most SEM microscopes detect two types of electrons, namely back-scattered electrons (BSE) and secondary electrons (SE). The former are produced due to elastic scattering of beam electrons from the atomic nucleus of the sample and are derived from the deeper parts of materials (100–1000 Å); the latter come from the sample and are produced as a result of bombarding of the surface atoms (10–300 Å) by beam electrons [21,31]. The detectors used for BE (passive detectors, scintillation detectors, semiconductor BSE detector) and SE (Everhart–Thornley detector, through-the-kens electron detectors) were described in [21]. Detection of the BSE signal produces images, in which contrast depends on relative differences in atomic numbers, in contrast to secondary electrons detection mode, which provides information only about the topography of the specimen. In general, the brighter the microarea, the higher its atomic mass. In contrast, the darker microareas indicate lower atomic mass, or resin-filled porosity. Thanks to these signals, the determination of phase composition can be postulated.

The cross-section sample for SEM observation was prepared by a ceramographic technique. First, the sample was embedded in an ambient cure two-component epoxy resin. Then, the resin-embedded cross-sectioned sample proceeded with rough grounding and fine polishing. The impregnated sample was coated with carbon in a vacuum to enable the charge transfer of SEM beam electrons at the ceramic, electrically isolated specimen. Finally, the sample was mounted on the SEM microscope table using carbon tape. 

For the quantitative analysis of the image, proper preparation of the sample is crucial [32]. Additionally, the number of images captured should be enough to retrieve the representative information, and will be greater for higher-magnification photographs. Based on experience, the approximate number of images that should be taken to obtain reliable information on a sample is 20–40, which, specifically, depends on the analyzed sample (larger for anisotropic materials). The average time of such analysis, together with EDS measurements, is 1–2 h. 

**Figure 2:**
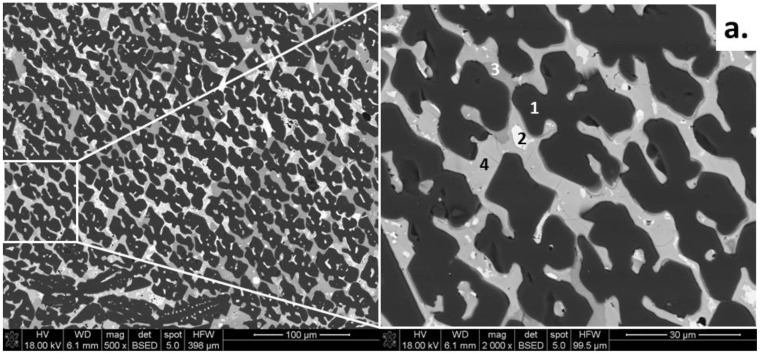

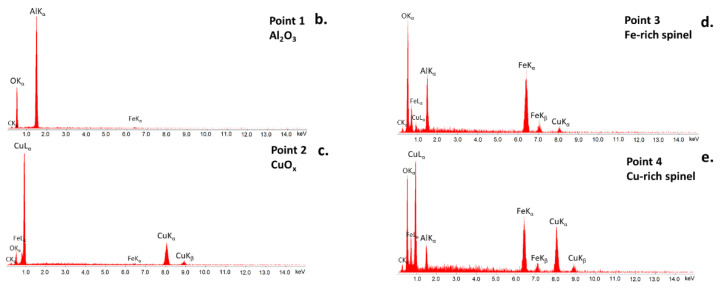
(**a**). SEM microstructure image of ceramic high-temperature material together with EDS spectra in points 1–4 (**b**–**e**).

Figure 2a presents a greyscale SEM microstructure image of the studied, melted ceramic material, which is composed of four distinct phases, determined based on the chemical EDS analysis. The darkest phase, which created a specific pattern, is Al_2_O_3_ with a slight amount of Fe (0.9, point 1). Between alumina grains, three phases of different intensities of grey are observed. The lightest-color phase is copper oxide, CuO_x_ (point 2). The rest phases, lighter-grey (point 3) and darker-grey (point 4), are spinel compounds of different stoichiometry, namely, Cu-rich and Fe-rich spinels, respectively. The EDS spectra for the discussed points are presented in Figure 2b, and the results are included in Table 1.

The SEM microphotograph, saved in lossless *tiff* format, was subjected to image analysis by using traditional methods based on stereology as well as an automated method based on the developed algorithm. For the traditional methods of linear and planimetry, the free and open-code software *ImageJ* was used [9]. The automatic algorithm for the simultaneous recognition and measurements of phases present in the studied sample was developed using *Aphelion* software ver. 4.4.0.

## 3. Traditional Methods of Image Analysis

Stereology covers the number of methods developed for the description of 3D objects based on 2D images [33]. The selected methods, especially planimetry owing to its usefulness, were included in standards, e.g., American Society for Testing and Materials [34] for the estimation of average grain size in all single-phase materials, or International Organization for Standardization [35] for the estimation of grain size in Cu and Cu alloys, and other applications [36]. Using stereological-based methods, the volumetric proportions between coexisting phases can be determined using only fragmental information based on a flat cross-sectioned sample. A reliable analysis requires a random area, using material that is located uniformly within the volume of the material and without the privileged direction of orientation [37]. 

Traditional stereological methods, including planimetry, linear analysis, or point counting, are used to obtain quantitative information about the objects distributed in the material. Specifically, the global parameters are determined. They describe relations between selected features and the entire analyzed space, including the volume share of the selected element in the material (V_V_), the surface area share of cross-sections (A_A_), and linear its share (L_L_). According to the Cavalieri–Hecquert principle, the global parameters are Equation (1) [33,38].

V_V_ = A_A_ = L_L_ (1)

### 3.1. Linear Analysis

The linear analysis is based on the secant of known length on the analyzed microstructure image followed by the determination of the sum of the chords belonging to this secant, covering the interest phase. The linear share is the ratio of the sum of chords to the length of the secant. A specific number of secants is applied for the analyzed image in order to reduce the uncertainty. The quantity information is obtained by dividing the sum of the chords cutting out the interest phase by the total length of the secant (2).

(2) $$L_{L}\left(P\right)=\frac{\sum_{i=1}^{n}\sum_{j=1}^{m}c_{ij}}{n\cdot l}$$

where:

c—the length of the chord,

n—the number of secants,

m—the number of the cut phase in the following *i*-measurement,

l—the length of the secant.

Based on the Cavalieri–Hecquert principle (1) and the calculated surface area of the interest phase (P1, P2, P3. or P4), the volume share of the specific phase in the material is equal to its linear share at the cross-section, namely V_V_ = L_L_ [33,38].

### 3.2. Planimetry 

Planimetry is based on the measurement of the surface area of the interest phase, via summing the pixels corresponding to this phase, extracted out of the rest phases by binarization. The quantity information is obtained by dividing the surface of the phase by the total surface of the image (3).

(3) $$A_{A}\left(P\right)=\frac{\sum_{i=1}^{n}A\left(P\right)}{A\left(A\right)}\;$$

where:

A(P)—the total surface of the phase at the image,

A(A)—the total surface of the analyzed image.

Based on the Cavalieri–Hecquert principle (1) and the calculated surface area of the interest phase (P1, P2, P3, or P4), the volume share of the specific phase in the material is equal to its surface share at the cross-section, namely, V_V_ = A_A_ [33,38].

## 4. Operations in Computer Image Analysis

**Figure 3:**
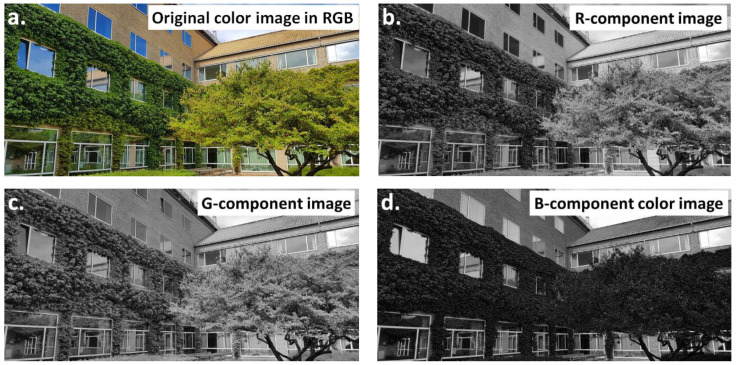
Comparison of (**a**) the color image in the RGB model, and images formed by decomposing the image into (**b**) R, (**c**) G, (**d**) B components.

**Figure 4:**
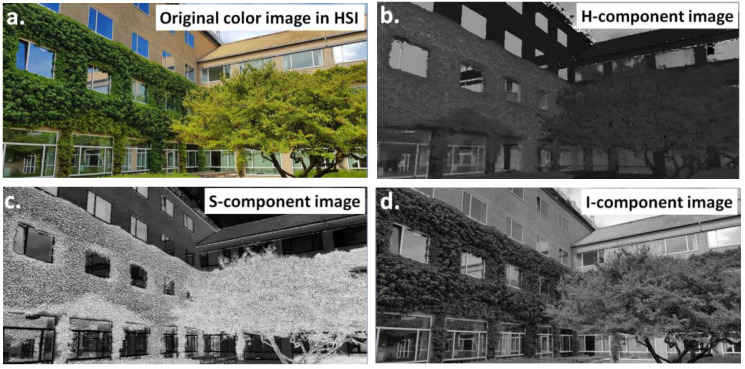
Comparison of the (**a**) color image in the HSI model and images formed by decomposing the image into (**b**) hue (H), (**c**) saturation (S), and (**d**) intensity (I) components.

Different color models, also called color spaces, are commonly used in image analysis. Using a color space, it is possible to define a specific combination of color models and their representation functions. The identification of a color space permits the automatic identification of the associated color models. During image processing, one of the important steps is the selection of the color model. Several color models were developed, as presented in Table 2. They can be used independently of the desired results, as shown by Figure 3 (RGB) and Figure 4 (HSI).

One method of image processing is converting an original color image into a grey one (0–255 grayscale [39]; Figure 1, middle), followed by analysis. This simple and quick transformation can extract information that can be used in the subsequent steps of the algorithm. However, it simultaneously causes a loss of information in the individual components. Alternatively, binarization of the color image can be performed for one individual component of the image, which permits obtaining the binary image directly from the color image (Figure 1, right).

If object detection on a grey image is not recommended or if a determination of the binarization thresholds for a color image is problematic, the RGB image can be split into components. This transformation produces three multi-shade images, representing the red (R), green (G), and blue (B) components, respectively (Figure 3a–d). Then, an individual image can be subjected to further transformations, such as object detection.

In some cases, the RGB model does not permit the detection of objects effectively. This results from insufficient color differences between the objects and the background, in terms of the values of the individual color components. Therefore, it may be useful to perform a color transformation from the RGB model to the HSI model. The result of this transformation is an image stored in the HSI model, as shown in Figure 4a–d. In this method, the color image is split into its components and the differences between the images are analyzed. Even if there is no significant difference in the share of the individual RGB components, the differences will be visible in the share of the components of saturation, intensity, and brightness of colors. The most suitable component can be selected for further transformation and detection.

**Figure 5:**
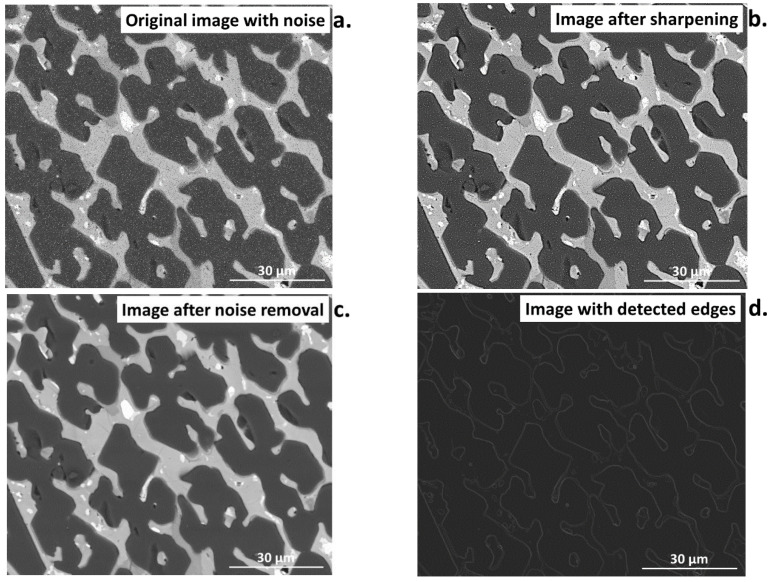
Comparison of the (**a**) input image with added noise, and images (**b**) after sharpening, (**c**) after noise removal, (**d**) with detected edges in the image without noise.

Noise is commonly encountered during image acquisition. This can make analysis difficult or, in some cases, impossible. For this purpose, several filter tools have been developed to reduce the presence of noise. However, special caution should be taken during using a filter for noise reduction to avoid the introduction of undesired changes to the sourced image. Otherwise, this can cause irreversible losses in the information contained in the image [39]. Filters are mainly used for sharpening, blurring, or edge detection [40], as presented in Figure 5a–d.

**Figure 6:**
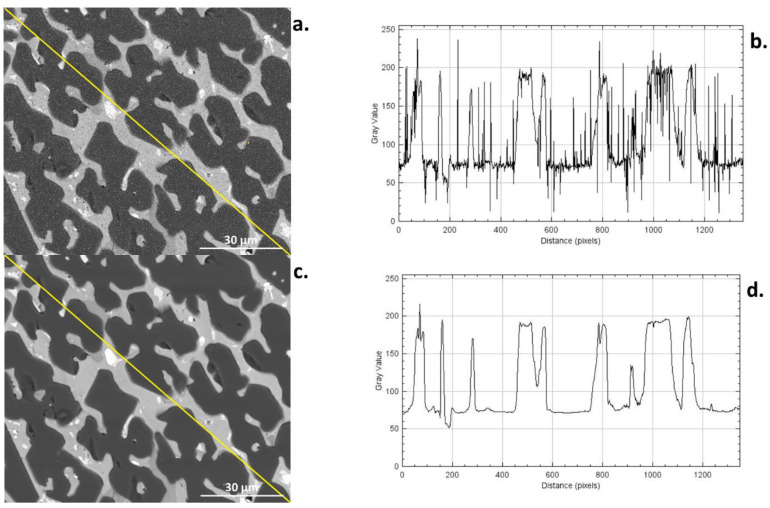
Comparison of the image and noise with its profile (**a**,**b**) vs. the image after noise reduction and its profile (**c**,**d**); the yellow line shows the test site for creating the profile.

Filters can help significantly in image preparation, e.g., if the image was registered with a signal. This can be evidenced by comparing the profile of the image with noise (Figure 6a,b) to the image after noise reduction (Figure 6c,d) using the median filter [39]. The profile after filtering is significantly smoothed compared to the image with noise. This is an ideal operation for inhomogeneous noise with varying degrees of grey. Sharpening filters should be avoided when performing quantitative image analysis as they can introduce additional noise, despite the improvement in the quality of the image. 

**Figure 7:**
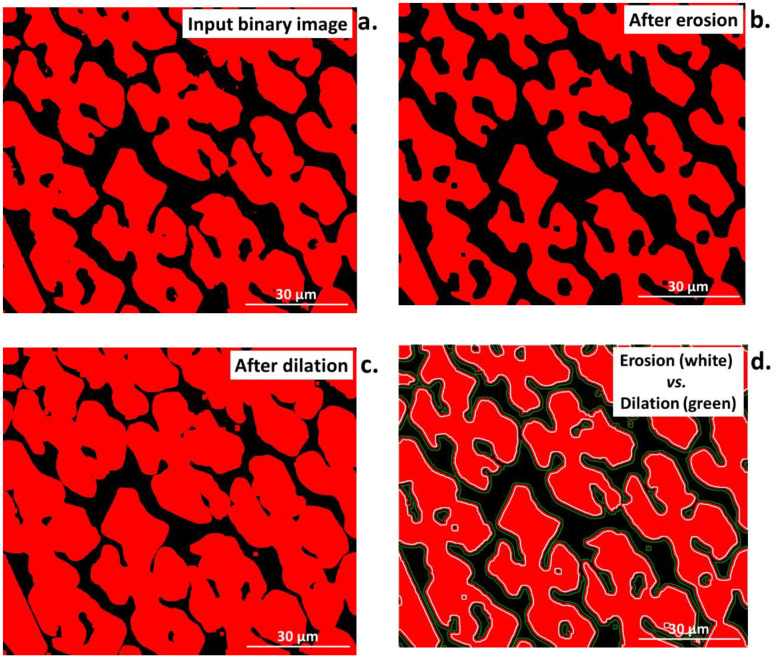
Comparison of the (**a**) input image after Otsu binarization to images after (**b**) erosion, (**c**) dilation, and (**d**) erosion (white lines) and dilation (green lines) superimposed on the input binary image.

Morphological operations belong to the most important operations in the analysis of the digital image, as, combined with other operations, they allow for complex image transformations to extract desired information. Morphological operations can be described as filters that are distinguished from classical filters by their selectivity [3,33,39]. In this case, the specific points are subjected to transformation. A structural element, called a pattern or a template, plays a role in every morphological operation. The general scheme of the algorithm for morphological operations can be divided into three stages. First, a center point to each point in the image is applied. Then, the configuration of points is checked to confirm that it is identical to one in the pattern. Finally, the operation according to the given transformation is performed [3]. Four morphological operations are mostly used, namely erosion, dilation, opening, and closing (Figure 7a–d).

Erosion permits the removal of isolated points, small particles, and narrow spears from an image, as shown in Figure 7b. In addition, it smooths the edges of objects in an image and reduces their surface edges. Erosion can also lead to the division of the objects into several smaller ones, so it can be used to divide connected particles.

Dilation is the inverse transformation to erosion, also called a maximal filter. The characteristic feature of dilation is that it closes small holes (fills the gaps in objects) [3,33]. As result, the area of each object in the transformed image is greater than in the input image, as shown in Figure 7c.

**Figure 8:**
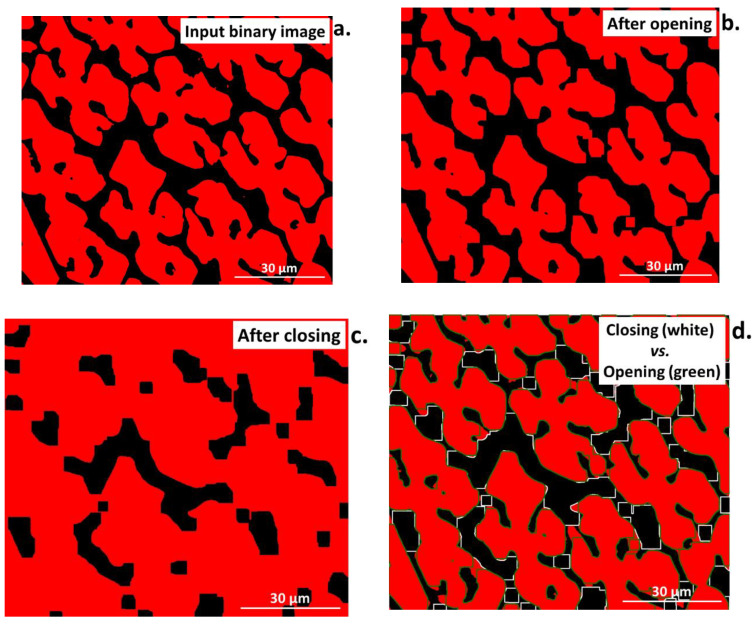
Comparison of the (**a**) input image after Otsu binarization to images after (**b**) opening, (**c**) closing, and (**d**) closing (while lines) and opening (green lines) superimposed on the input binary image.

Opening and closing are more complex operations, combining erosion with dilation (Figure 8a–d). In the opening operation, the erosion is followed by dilation. However, it uses the same pattern (structural element) and maintains the same operation size in both steps. First, erosion breaks up the thin lines in the input image that connect objects. Then, dilation permits the approximate recreation of these connections [3,33]. As a result, the separation of elements will be obtained in the output image, as shown in Figure 8b. The characteristic feature of the opening operation is that it removes small elements and details without changing the size of the main part of the image.

The closing operation is the reverse of the opening one. It includes dilation followed by erosion. The main role of closing is to fill in the narrow elements in the image, such as indentations or small details. Similar to opening, closing does not change the size of the image [33]. The results of the closing operation can be seen in Figure 8c and the comparison between opening and closing is presented in Figure 8d. 

Binarization is one of the most significant operations in image analysis [3,39,41]. It facilitates the analysis and conducting of measurements at the images, especially in terms of percentage quantification of the objects relative to the rest of the image, determination of the object parameters (areas, perimeters, etc.), and displaying them on screen. Numerous binarization methods have been developed so far, e.g., applying an upper threshold, a lower threshold, and two thresholds. Moreover, various automatic binarization methods are used that determine threshold values based on the analysis of selected image features. The Otsu or entropy thresholding method is an example of the automatic threshold algorithm, based on the histogram of grey-level distribution.

**Figure 9:**
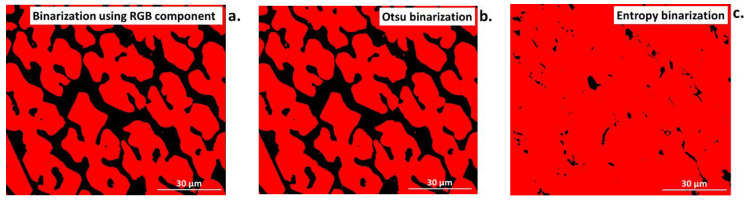
Comparison of various binarization methods, (**a**) using the blue component for RGB, and using automatic threshold methods (**b**) Otsu, (**c**) entropy.

**Figure 10:**
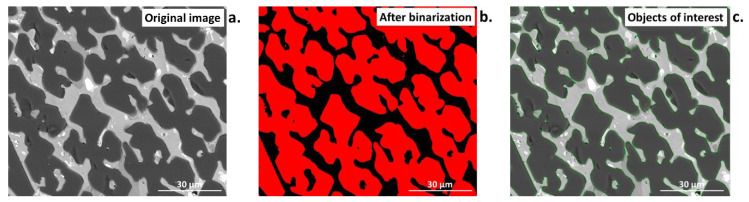
Comparison of (**a**) the original microstructural image of ceramic material with the image (**b**) after binarization, (**c**) comparison of images with marked objects of interest (green) permits for the visual assessment of the quality of the binarization.

As can be seen from Figure 9a–c, every individual method of binarization produces different results. Otsu binarization (Figure 9b), which is a histogram-based method, transforms a grayscale input image into a binary image by minimizing the weighted sums of the variances of two classes, namely foreground objects and background [41]. This method belongs to simplest and most successful; however, it may lose some important details. The entropy threshold method (Figure 9c) provides a set of regions from the input image, by applying automatic thresholding based on the entropy computation of the histogram. This method, due to fixed threshold values, has problems with images of unimodal histograms and may lose information for small, low-contrast target objects [41]. In all cases, to acquire information about the reliability of the selected binarization method as well as to avoid errors, the object of interest in the binary image (Figure 10b,c) should be compared to the original, before binarization (Figure 10a).

Binarization by automatic thresholding has both pros and cons compared with manual thresholding. The advantage is that automatic binarization does not require the selection of the range of threshold for a particular object, as it occurs automatically, releasing object and background distribution. In the case of entropy automatic binarization, for each threshold, the *t*-value (between image-min and image-max) and two probability distributions (object and background distribution) are derived from the original grey-level distribution image [41]. As result, two entropy values are calculated. For a small number of studied objects and a large series of photographs, the use of the automated method is an appropriate solution if the same image quality is provided. However, when the number of interest phases increases or there are slight differences in the quality of the images, it may result in different detection of objects, e.g., for sample 1 the range needed for detection of a studied phase is 0–100, while for sample 2 it is 0–120 for the same object. Overall, the choice of binarization method shall be adapted to the studied image and object of interest; however, in the former case, the manual (interactive) method will be more appropriate as it permits precise adjusting of the range of threshold [39]. Automatic binarization works best for images with that which significantly differ in contrast, such as bright objects on dark backgrounds or the opposite [33]. Nevertheless, binarization alone is not a sufficient operation before image analysis, and in most cases requires additional operations, such as morphological transformations.

## 5. Results of the Quantification Analysis of the SEM Image

### 5.1. Traditional Stereology-Based Methods

In this work we used linear and planimetry methods. The measurements were performed on a digital SEM image. The digital image was analyzed using *ImageJ* software. Twenty-five lines of the same length were applied in the linear analysis. For planimetry analyses, before performing the binarization, the image was first filtered using a median filter, then transformed to an 8-bit image (256 of grey), normalized, and finally transformed to a 1-bit image using interactive thresholding.

**Figure 11:**
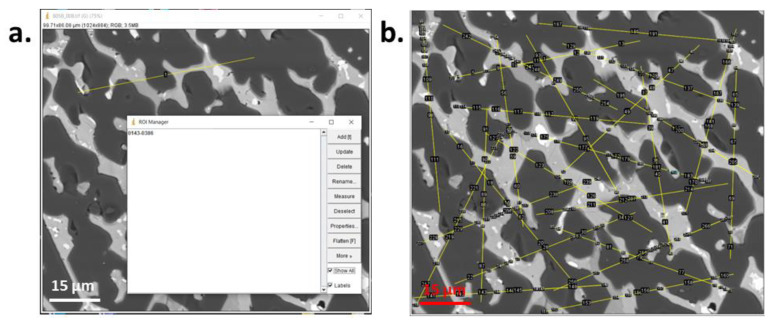
Windows of *ImageJ* during the linear analysis of the image, (**a**) using ROI Manager for adding the secants and chords, (**b**) with all secants and chords.

**Figure 12:**
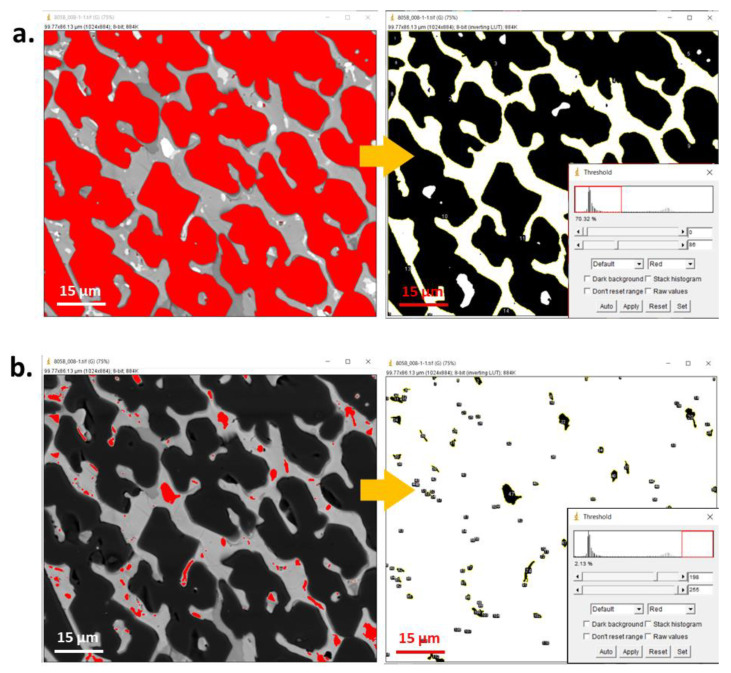

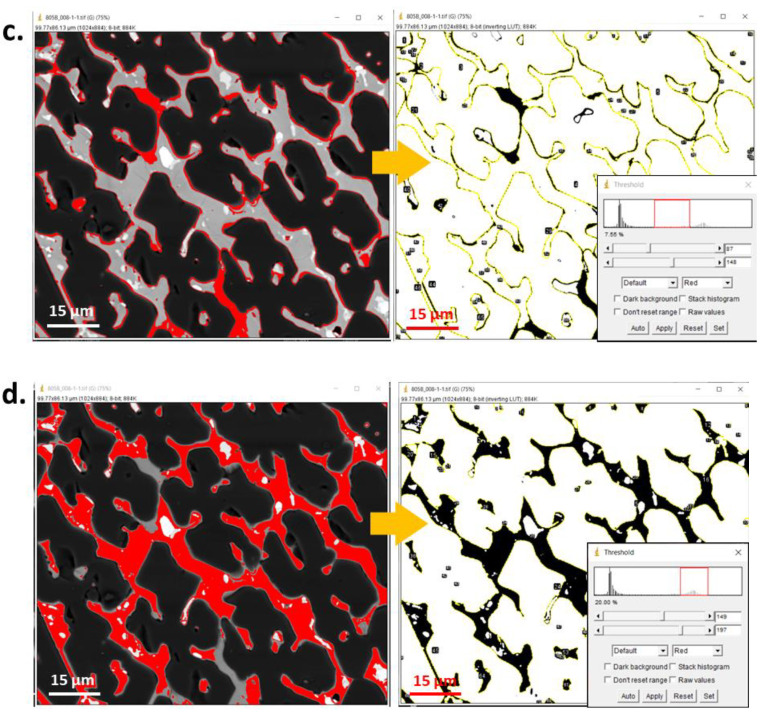
Window of *ImageJ* during binarization (by manual thresholding) and planimetry analysis of phases (**a**) P1, (**b**) P2, (**c**) P3, (**d**) P4.

The results of linear analysis on a grey SEM image of the studied material are presented in Figure 11a,b, while the result of the planimetry is presented in Figure 12a–d. The quantitative information extracted from both analyses are included in Table 3.

As can be seen from Table 3, both methods produced similar results. The greatest agreement was obtained for the P2 phase, which was CuO_x_, randomly distributed in the matrix, at the level of 2.7% and 2.1% for the linear and planimetry methods, respectively. Additionally, a similarly small difference between results was determined for the P1 phase, Al_2_O_3_, which constituted 70.6% and 71.4% for the linear and planimetry methods, respectively. The light-grey Cu-rich spinel phase P4, which occurred as a continuous phase, showed a higher discrepancy in both methods, of 24.6% (linear) and 21.6% (planimetry). The greatest difference was determined for the dark-grey Fe-rich spinel phase P3 of 1.9% (linear) and 8.6% (planimetry). The difference results from detecting the edges of the Al_2_O_3_ grains as the P3 phase, due to the same pixel values.

### 5.2. Automated Method

**Figure 13:**
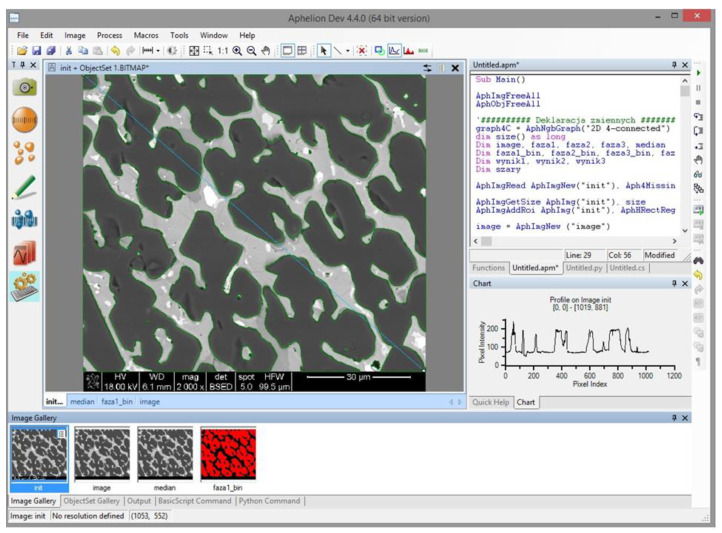
User interface of *Aphelion* during the automated analysis of an SEM image of high-temperature ceramic material.

*Aphelion* software was used for the development of the algorithm for automated image analysis. The user interface of *Aphelion* during analysis is presented in Figure 13. The software is equipped with the necessary modules to perform individual operations and permits the simultaneous detection and percentage amount measurements of the objects present in the SEM image. In addition, all the data produced during analysis can be saved as a macro command in the Visual Basic language. 

**Figure 14:**
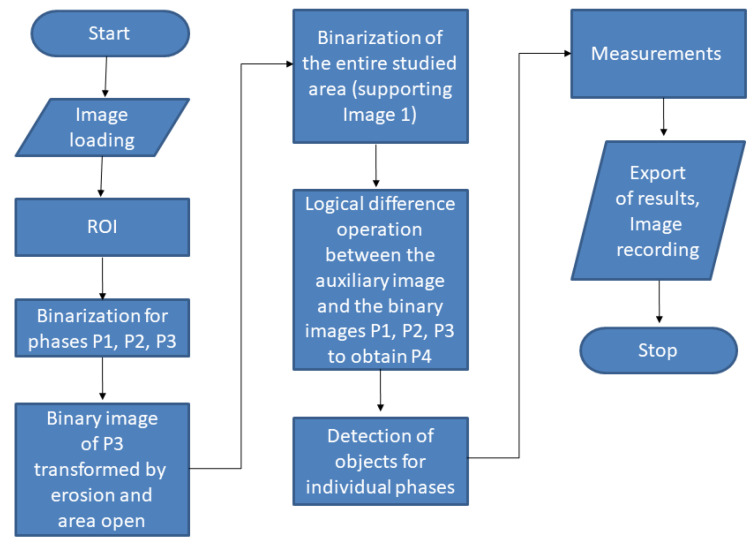
Flowchart summarizing the developed algorithm for image analysis.

The goal of the developed algorithm was to automatically detect the four phases present in the multiphase material, namely P1, P2, P3, and P4 (previously determined using SEM/EDS on cross-sections), color every individual phase as separate, and finally, measure its percentage 2D surface area relative to the total image area. A schematic presentation of the developed algorithm is depicted in Figure 14.

**Figure 15:**
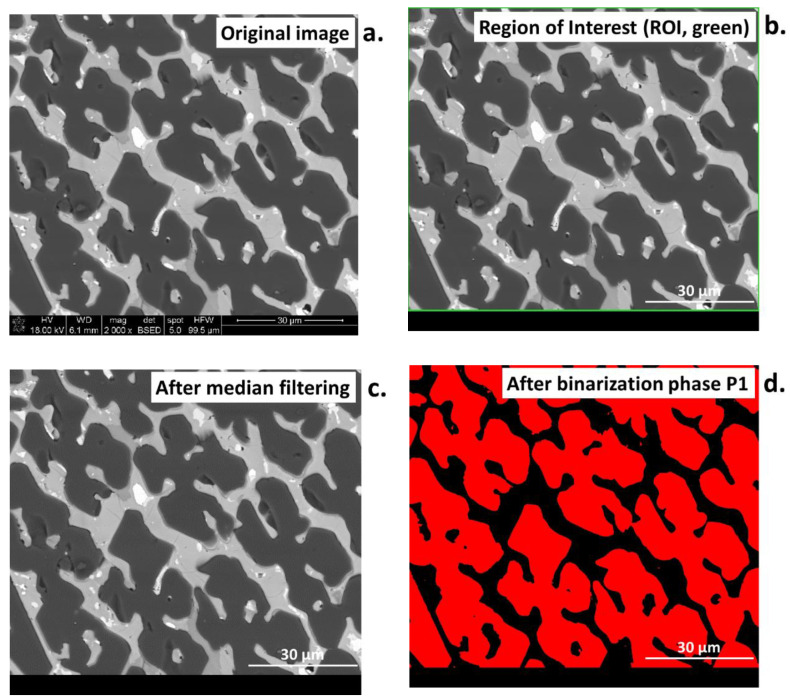

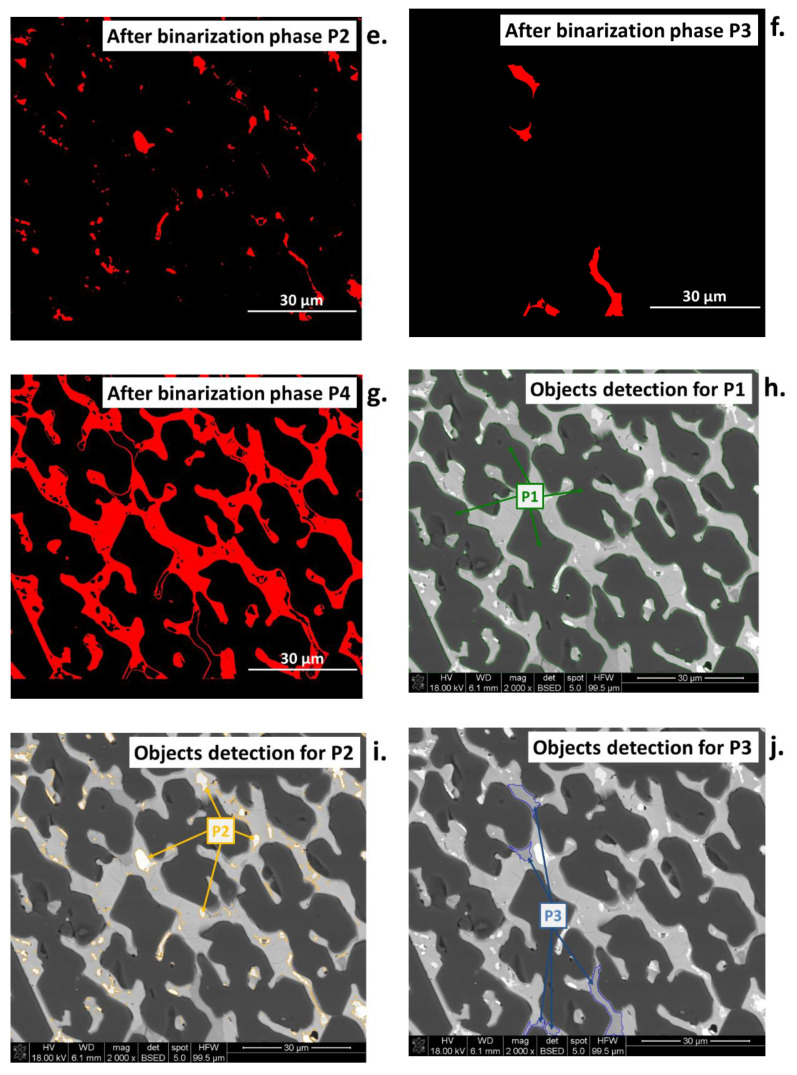

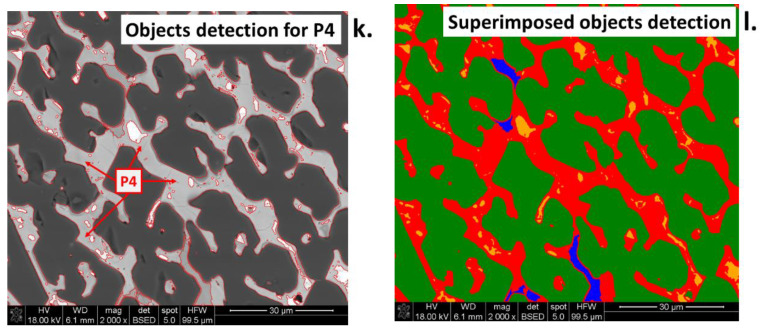
(**a**–**l**) Images presenting the steps of the algorithm for image analysis of high-temperature ceramic material.

Figure 15a–j show the output images from the following steps of the image analysis. In the first stage, the input image (Figure 15a) is marked with the region of interest (ROI. Figure 15b) to remove the black bar along the bottom that contains metadata of the SEM analysis. Only the ROI is taken for the image analysis, which is then subjected to median filtering to remove minimal noise (Figure 15c). Subsequently, the binary images obtained by thresholding are generated for every individual object of interest, namely phases marked as P1, P2, and P3 (threshold selected using a profile; Figure 15d–f). The fourth object—P4—is determined by first detecting the ROI analyzed area and subsequently applying a log difference between ROI and the previously detected objects P1, P2, and P3 (Figure 15g). Additionally, the alternating operations of erosion, areaopen (a built-in function in *Aphelion*), erosion, and areaopen were applied, which allowed the removal of the lines of P1 phase grain boundaries (treated the same as P3) and final detection of the P3 phase. The next step was the presentation of every detected phase in a separate image (Figure 15h–k). Finally, all the detected phases were superimposed on the same image, marked in different colors for better and complete visualization of the results (Figure 15l).

The percentage content of each phase was calculated based on the corresponding surface area with reference to the total surface area of the analyzed image, according to Equation (4), and was presented in Table 4.

Vv(P) = A(P)/A(A) (4)

where:

A(P)—the surface area of the analyzed object P1, P2, P3, or P4;

A(A)—the surface area of the total sample.

**Figure 16:**
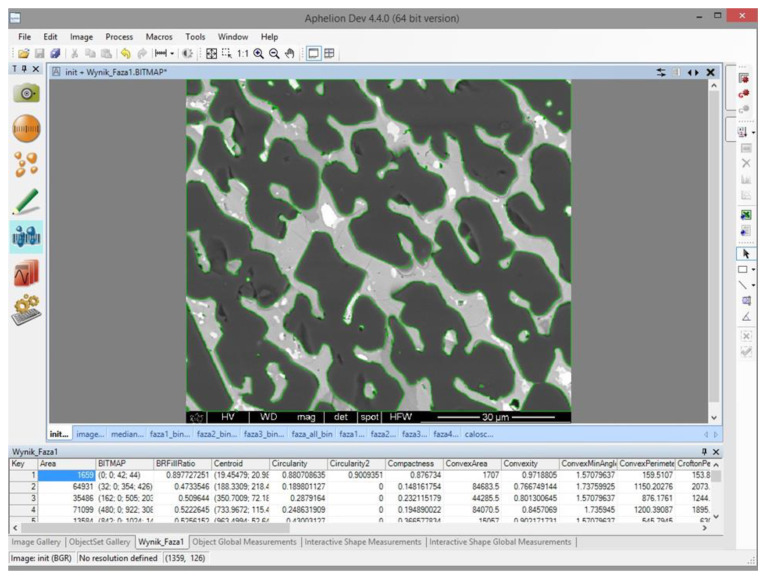
User interface of *Aphelion* during measurements of the P1 phase.

**Figure 17:**
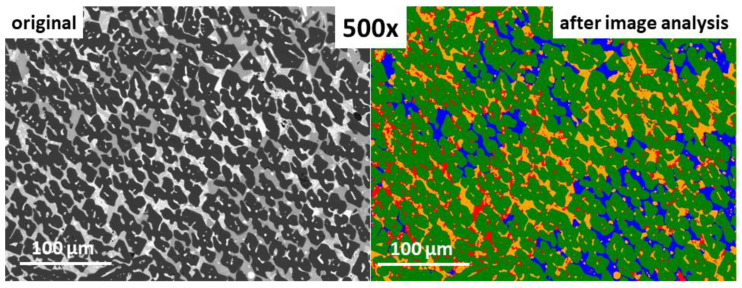
500× magnified original SEM image (**left**), and image the after analysis using the developed algorithm (**right**).

The window of the software during the image analysis was showed in Figure 16. The superimposed image with all the detected phases, in magnification 500× was demonstated in Figure 17. The results of image analysis can be exported to CSV and Excel formats. The parameters given by the software involve area, volume fraction, Crofton perimeter, etc., are attached in Table 5, and can be further used for statistical purposes. The Crofton perimeter is an estimate of object perimeter (more complex than the four-point connections-based neighbor-analysis <*Perimeter*> and results in high accuracy. Additionally, it provides a more accurate estimate of the Euclidean object perimeter and is less sensitive to object orientation compared to *Perimeter* [39].

**Table 4:** Percentage distribution of analyzed phases P1, P2, P3, and P4 in the image, corresponding to Figure 15l (2000×) and Figure 17 (500×).

Phase Name *	Amount of Phase, %	
Magnification	2000×	500×
P1 (green)	66.3 ± 0.1 *	69.3 ± 0.1
P2 (orange)	10.7 ± 0.4	2.8 ± 1.3
P3 (blue)	9.9 ± 0.2	1.2 ± 2.1
P4 (red)	13.1 ± 0.2	26.7 ± 0.1

* relative error.

## 6. Discussion

In this work, we showed the results of image analysis conducted using two approaches: traditional stereology-based and automated techniques. Linear analysis and planimetry were applied as a traditional route, while an algorithm was developed to automatically process and analyze the SEM image of ceramic multiphase material. 

Image analysis is most reliable for the non-porous, homogeneous material. However, common real materials are inhomogeneous and contain defects such as intergranular or inside-grain porosity. An example is slag samples from non-ferrous metallurgy processes [42]. The traditional stereology-based image analysis for such materials would need analysis of multiple images and would be extremely time-consuming. Thus, for this kind of material computer, image analysis utilizing algorithms for the automated detection and measurements of the material components is most promising and needed.

**Figure 18:**
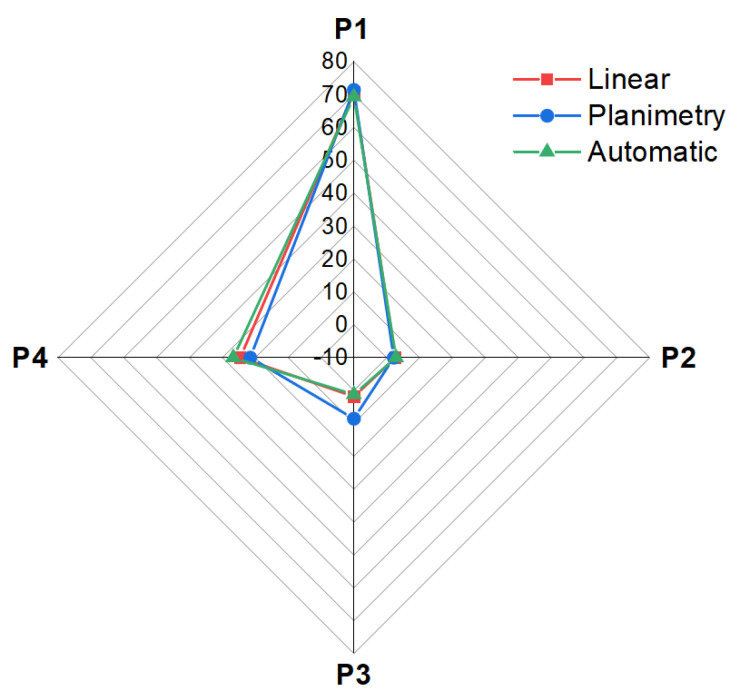
Spider chart presenting the results of image analysis obtained using traditional (linear and planimetry) and automated methods.

**Figure 19:**
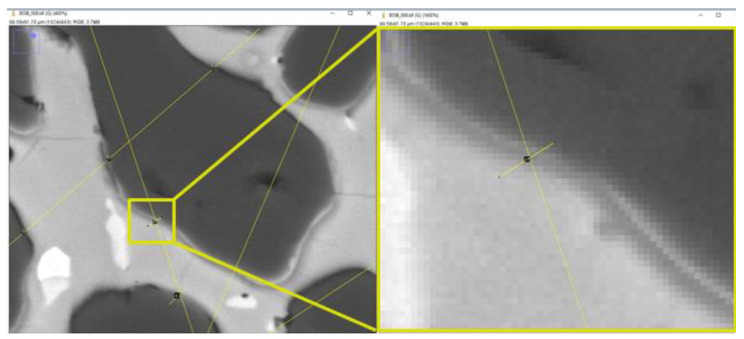
Magnified SEM image during linear analysis showing the source of the error resulted from blurred grain boundary.

The results of the SEM image analysis conducted in this work using traditional and automated methods were found to be in good agreement, as seen in Figure 18. The dispersion between phase amounts using traditional (linear) and automated methods was below 1.5% for P1–P3 and 2% for P4 (Table 3 vs. Table 4). The dispersion of results for phase P3 (dark-grey phase in the matrix) between linear and planimetry methods results from counting the pixels of P1 phase at grain borders, which were equal as it comes to their value to pixels for the phase P3 (Figure 19). The analyzed image contained phases of different mechanical properties, with the most abundant high-hardness Al_2_O_3_ phase (9 on the Mohs scale) distributed among softer spinel phases (8 on the Mohs scale) [43] and tenorite CuO (3.5 on the Mohs scale). This produced blurred grain boundaries of the P1 phase, which appeared brighter and had pixel values the same as the P3 phase, causing the P1 boundaries to be treated by the algorithm as P3.

**Figure 20:**
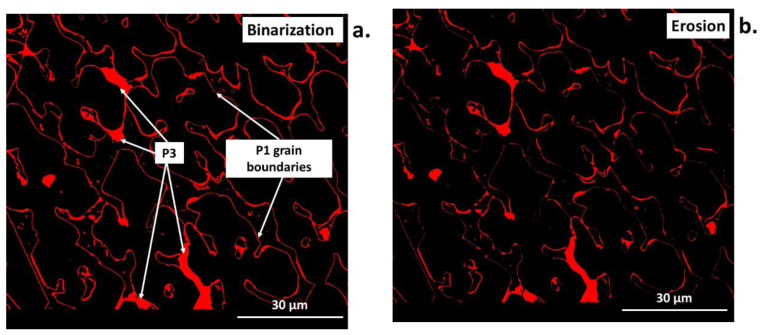

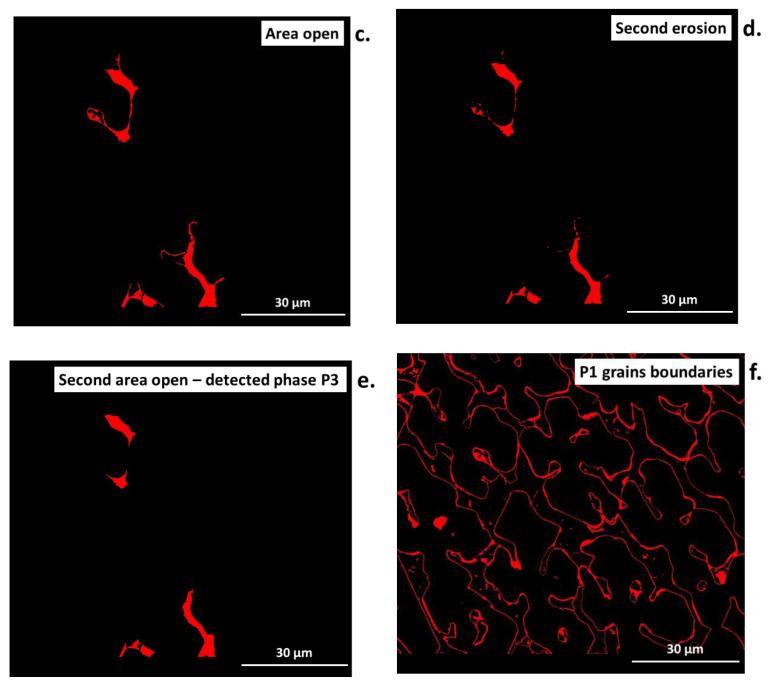
SEM image during automated analysis for P3 phase: (**a**) after thresholding, (**b**) after thresholding followed by morphological transformations (**c**–**f**).

The challenge of the algorithm in distinguishing the binary image between the P3 phase and the grain boundaries of the P1 phase (Figure 20a–f) was resolved using the combined action of morphological operations. The following sequence of morphological operations was applied to solve the issue, namely erosion, area open, erosion and, again, area open. This resulted in the correct P3 phase detection. For the detection of only the Flines representing the P1 grain boundaries, the algorithm used the logical difference between the original binary image (Figure 20a) with the previously detected P3 phase (Figure 20e).

The automated computer image analysis enables the simultaneous phase detection of multiple-phase material on the analyzed digital SEM image, and allows for measurements of their amounts. The quantity measurements are conducted on the binary images, so images are reduced to 1-bit color depth via thresholding. Thus, binarization plays a key role in image analysis and may be the source of large errors. The results obtained in this work by the automated method confirm those obtained by linear and planimetry belonging to the traditional methods. Often, converting an initial color image in the RGB model into another one, such as HSI, helps to make the algorithm simpler and the results more satisfactory.

Numerous obstacles may arise during development of the algorithm for automated image analysis. Frequently, noise causes difficulties in proper object detection since small elements are treated as searched objects (Figure 4a). Another obstacle is a shadow, defined as uneven background illumination. A shadow can cause uneven illumination of objects and is produced during image acquisition, especially in the case of optical microscopy. As a result, the image is brighter in the middle and darker at the border of the field of view. This is a common problem in microscopic images and leads to incorrect qualitative descriptions of the objects being analyzed at a given time. The human eye does not notice subtle and minimal differences in background brightness. Approximately 40–50 shades of grey are recognized by the human eye [39,44]. The shading effect leads to difficulties in detecting objects correctly, especially using algorithms with automatic thresholding [23,45].

Another aspect that can affect automated object detection is the choice of image analysis method, i.e., algorithms based on human knowledge and experience or based on machine learning or neural networks. Automated detection is faster and more accurate, while human-made ones, based on experience in creating image analysis solutions, are simultaneously more able to detect errors and provide better control over the final result [46,47]. Nevertheless, the code should be transparent and as simple as possible, built by applying the best initial knowledge and experience of a human.

Performing reliable image analysis strongly depends on the sample preparation procedure as well as the conditions of SEM analysis, which both should be repeatable to obtain sharp boundaries between microstructural objects. Numerous factors impact the quality of SEM images and the conditions of their repeatability. The depth of field of a scanning electron microscope is dependent on many factors of both physical and microscope construction nature, including the diffraction of electrons, the diameter of the aperture, magnification, and the working distance (which impacts spot size and aperture angle). These wider aspects can be developed in future works.

One of the most important aspects, in terms of the industrial application of automated image analysis based on dedicated algorithms, is the time required for image analysis. Traditional techniques for the quantitative determination based on stereological principles (using parameters V_V_, A_A_, L_L_) are universal and efficient but, simultaneously, time-consuming. In this work, the time to perform the computer-aided linear analysis together with the analysis of the results was 170 min, but 60 min for the planimetry (the classical manual method would take at least 50% longer). In contrast, the total time for the analysis after the development of the algorithm was less than 5 s. Thus, the application of modern software for image analysis, such as *Aphelion*, facilitates the development of dedicated algorithms for precise, more user-friendly, and faster image analysis. 

## 7. Conclusions

In this work, a digital SEM image of four-phase high-temperature ceramic material from the Cu-Al-Fe-O system was subjected to image analysis to obtain quantitative data on the coexisting phases using traditional (linear, planimetry) and automated method. The results of stereology-based methods and automated methods utilizing the developed algorithm of image analysis produced comparable results with a maximum 2% dispersion, requiring 5 s for the automated method compared to 60 min for classical planimetry. This shows that automated methods of image analysis can be successfully used for precise, effective, and fast SEM image analysis, and can be widely applied for the characterization of microstructure and tailoring of the properties of ceramic high-temperature materials and other similar materials.

## Figures and Tables

**Table 1:** EDS analysis of microareas of high-temperature ceramic material corresponding to Figure 2.

Point	Phase	Chemical Composition, mol. % *		
		Cu	Fe	Al
1	Alumina Al_2_O_3_	-	0.9	47.0
2	Copper oxide CuO_x_	67.3	1.8	0.6
3	Fe-rich spinel (Fe,Cu)(Fe,Al,Cu)_2_O_4_	2.6	31.9	13.0
4	Cu-rich spinel (Cu,Fe)(Cu,Fe,Al)_2_O_4_	30.7	19.4	7.7

* The rest (to 100%) is oxygen.

**Table 2:** Selected color models and their designations.

Name	Color Space Definitions
RGB	Red, Green, and Blue
HSI	Hue, Saturation, and Intensity
HSV	Hue, Saturation, and Value
YUV	Luminance and Chrominance

**Table 3:** Results of traditional image analysis corresponding to Figure 2 (linear) and Figure 3 (planimetry).

Phase Name	Phase Amount, %	
	Linear	Planimetry
P1 (darkest)	70.6	71.4
P2 (lightest)	2.7	2.1
P3 (dark grey)	1.9	8.6
P4 (light grey)	24.6	21.6

Estimated error: linear ±2.0, planimetry ±1.0.

**Table 5:** Two-dimensional parameters obtained as a result of the automated image analysis using *Aphelion* software.

Parameter	Description
Pixel Count	Number of pixels making up the object
Height	The difference between an object’s highest Y coordinate and its lowest Y coordinate
Width	The difference between an object’s right X coordinate and its left X coordinate
Centroid	The average position of all pixels in an object expressed as a pair of x, y coordinates (i.e., the center of mass of the object)
Major Axis	Angle in radians from the X-axis of the principal axis of inertia. This object attribute gives the main orientation of the object to the X-axis.
BR Fill Ratio	The ratio between the area of an object and the area of its bounding rectangle. The bounding rectangle has the same orientation as the X.Y coordinate system of the image.
Perimeter	An estimate of the object perimeter based on the number of 4-connected neighboring pixels along the object boundary
Crofton Perimeter	Facility circuit estimate based on a more complex analysis than 4-connectivity
Compactness	An object attribute that is equal to 16.Area/Perimeter^2
Bounding Rectangle To Perimeter	The ratio between the perimeter of an object and the perimeter of its bounding rectangle, where the latter is oriented along the X, and Y axis. The perimeter measure used for this ratio is Perimeter, as described above.
Number of Holes	The number of holes in an object. A hole is one or more connected background pixels completely contained within an object.
Area	Facility area
Elongation	The absolute value of the difference between the inertia of the major and minor axes is divided by the sum of these inertias. The minor axis is defined as the axis perpendicular to the major axis.
Circularity	For a given object this attribute is equal to: $\frac{4\pi \times Area}{Crofton\;Perimeter^{2}}$
Intercepts	Several transitions from background to object in 0°, 45°, 90°, and 135° directions
Equivalent Diameter	Specifies the diameter of a circle whose area is equal to the area of the object
Convexity	This attribute is equal to the area of the object divided by the area of its convex hull
Perimeter Variation	The sum of the changes in direction between the boundary pixels where a change of 45 degrees equals 1, a change of 90 degrees equals 2, and a change of 135 degrees equals 3
Convex Min Angle	The minimum of the angles formed by adjacent pairs of line segments comprising a polygonal object boundary is given in radians
Symmetry Mean Difference	The average of the absolute values of the difference in length between the centroid and the two opposite boundary points of the object
Convex Area	The convex hull area of the object
Convex Perimeter	Circumference of the object’s convex hull using the Perimeter measure
Holes Area	A vector containing the surface area of the holes in the object
Holes Total Area	The total area of facility openings

## Data Availability

The data presented in this study are available on request from the corresponding author.
